# The effect of a decision aid intervention on decision making about coronary heart disease risk reduction: secondary analyses of a randomized trial

**DOI:** 10.1186/1472-6947-14-14

**Published:** 2014-02-28

**Authors:** Stacey L Sheridan, Lindy B Draeger, Michael P Pignone, Barbara Rimer, Shrikant I Bangdiwala, Jianwen Cai, Ziya Gizlice, Thomas C Keyserling, Ross J Simpson

**Affiliations:** 1 Division of General Medicine and Clinical Epidemiology, University of North Carolina, Chapel Hill, NC USA; 2 Center for Health Promotion and Disease Prevention, University of North Carolina, Chapel Hill, NC USA; 3 Gillings School of Global Public Health, University of North Carolina, Chapel Hill, NC USA; 4 Department of Biostatistics, University of North Carolina, Chapel Hill, NC USA; 5 Division of Cardiology, University of North Carolina, Chapel Hill, NC USA; 6 Cecil G. Sheps Center for Health Services Research, University of North Carolina, Chapel Hill, NC USA

**Keywords:** Decision support techniques, Medication adherence, Heart disease, Primary prevention

## Abstract

**Background:**

Decision aids offer promise as a practical solution to improve patient decision making about coronary heart disease (CHD) prevention medications and help patients choose medications to which they are likely to adhere. However, little data is available on decision aids designed to promote adherence.

**Methods:**

In this paper, we report on secondary analyses of a randomized trial of a CHD adherence intervention (second generation decision aid plus tailored messages) versus usual care in an effort to understand how the decision aid facilitates adherence. We focus on data collected from the primary study visit, when intervention participants presented 45 minutes early to a previously scheduled provider visit; viewed the decision aid, indicating their intent for CHD risk reduction after each decision aid component (individualized risk assessment and education, values clarification, and coaching); and filled out a post-decision aid survey assessing their knowledge, perceived risk, decisional conflict, and intent for CHD risk reduction. Control participants did not present early and received usual care from their provider. Following the provider visit, participants in both groups completed post-visit surveys assessing the number and quality of CHD discussions with their provider, their intent for CHD risk reduction, and their feelings about the decision aid.

**Results:**

We enrolled 160 patients into our study (81 intervention, 79 control). Within the decision aid group, the decision aid significantly increased knowledge of effective CHD prevention strategies (+21 percentage points; adjusted p<.0001) and the accuracy of perceived CHD risk (+33 percentage points; adjusted p<.0001), and significantly decreased decisional conflict (-0.63; adjusted p<.0001). Comparing between study groups, the decision aid also significantly increased CHD prevention discussions with providers (+31 percentage points; adjusted p<.0001) and improved perceptions of some features of patient-provider interactions. Further, it increased participants’ intentions for any effective CHD risk reducing strategies (+21 percentage points; 95% CI 5 to 37 percentage points), with a majority of the effect from the educational component of the decision aid. Ninety-nine percent of participants found the decision aid easy to understand and 93% felt it easy to use.

**Conclusions:**

Decision aids can play an important role in improving decisions about CHD prevention and increasing patient-provider discussions and intent to reduce CHD risk.

## Background

Promoting use and effective adherence to coronary heart disease (CHD) prevention medications is integral to improving preventive care and reducing healthcare costs [1-4]. However, it raises challenges for both patients and providers. Challenges fall broadly in two categories: those related to improving decision making and patients’ intent to initiate one or more similarly effective medications for CHD risk reduction, and those related to supporting adherence.

Challenges related to improving decision making include overcoming patients’ lack of knowledge about CHD risk factors [2,3], their CHD risk [4], and the potential benefits of risk reducing strategies [5-8]. They also include helping patients clarify their values for various heart disease prevention strategies [9,10] and communicate effectively with their provider to align their treatment priorities [11-24].

Challenges related to supporting adherence include fostering patients’ confidence and skills for adherence and marshaling adequate health system and social support to ensure the likelihood of patients’ success [14]. Most work to date has focused on these latter issues related to adherence with relatively little work focusing on improving patients’ decision making about initiation of medications [25,26].

To improve decision making, some have recommended use of decision aids. Decision aids are multi-media tools designed to convey health information, help patients clarify their values, choose health options that are consistent with their values and resources, and communicate their treatment priorities to their provider. [27] Systematic reviews have shown that decision aids improve patient knowledge and values clarity, and increase the likelihood of making decisions [27]. However, few decision aids have been studied in the adherence context [26,28-31]. Further, none to our knowledge has focused on the choice among several similarly effective medications to reduce CHD risk, helped patients to clarify their values and communicate their treatment preferences to their provider, or coupled decision-making with tailored messages to overcome barriers to adherence.

In this paper, we report on the secondary outcomes of a randomized trial of a CHD adherence intervention. We have already reported that the overall intervention (second generation decision aid plus tailored adherence messages to overcome barriers to adherence) is effective in improving self-reported adherence (+25 percentage points, 95% CI 8 to 42%) and reducing 10-year predicted CHD risk (-1.1 absolute percentage points, 95% CI -0.16% to -2%) at 3-month follow-up [32]. Now, in an effort to further understand how our intervention produced these effects, we examine the independent effects of the decision aid on patients’ knowledge, accuracy of risk perception, decisional conflict, values clarity, patient-provider interactions, and intentions for CHD risk reduction. We also examine intent for CHD risk reduction across the various components of the decision aid (education, values clarification, and coaching to discuss CHD risk reduction with the provider) and report on patients’ use and overall perceptions of the decision aid.

## Methods

### Overview

We conducted a randomized trial of a CHD adherence intervention called Heart to Heart at one university general internal medicine practice. Detailed methods of this trial and its main outcomes are described elsewhere [32]. A brief overview of the trial and details about the decision aid and measurement of decision making outcomes are provided below. Prior to study participation, participants provided written informed consent for study participation. The University of North Carolina at Chapel Hill’s Biomedical Institutional Review Board (IRB) approved all study procedures.

### Setting

We conducted our study in one university internal medicine clinic, which employs 93 providers (17 attendings and 76 residents) who were not part of our research team and eligible to participate in the study, allowing us to enroll their patients. Forty of these providers agreed to participate in the study and allow us to enroll their patients and 24 had patients who agreed to enroll in the study.

### Participants

Patients were eligible for participation in the study if they were presenting for care with an enrolled provider, were between 40-79 years old, had no prior history of cardiovascular disease, diabetes mellitus, or other serious medical condition that limited their life expectancy to less than five years, and were at moderate (6-10%) to high (>10%) risk of heart disease over the next 10 years based on a Framingham risk equation [33]. Detailed exclusion criteria are published elsewhere [32].

### Study procedure

After collecting baseline measures at an initial study visit, we centrally randomized patients to either the intervention or control (usual care) group and saw them for two additional study visits over 3 months. This paper focuses on data collected at the second or “primary” study visit.

At this visit, patients randomized to the intervention group presented 45 minutes early to a previously scheduled provider visit, viewed the decision aid, answered questions about intent for CHD risk reduction embedded after each decision aid component, and filled out a post-decision aid survey assessing their knowledge, accuracy of risk perception, decisional conflict, values clarity, and intent for CHD risk reduction. Patients randomized to the control group did not present early to their previously scheduled clinic visit and received usual care from their provider. Following the provider visit, a research assistant gave self-administered post-visit surveys to participants in both groups to complete. Post-visit surveys assessed participants’ discussions with their providers, and their intent for CHD risk reduction.

### Intervention

Our intervention consisted of two parts: a decision aid delivered prior to a provider visit (at the primary study visit) and a series of three tailored adherence messages delivered between the primary and follow-up study visits. In this paper, we focus on the independent effects of the decision aid, which includes three components: individualized risk assessment and education; values clarification; and coaching [32]. In contrast to our earlier CHD prevention decision aid [34], this second generation decision aid was professionally developed by the Communications for Health Applications and Interventions Core at the University of North Carolina at Chapel Hill (http://www.chaicore.com) and incorporates graphics and photographs to increase engagement with the information provided. It also includes values clarification and coaching in addition to education.

The individualized risk assessment and education component of our decision aid calculates a patient’s global risk of CHD events (e.g. angina, myocardial infarction, and death) in the next 10 years using a continuous Framingham equation [33] and then provides patients with individualized information about their global CHD risk, their personal risk factors, the pros and cons of pertinent CHD risk-reducing therapies (which may include aspirin, hypertension medications, cholesterol medications, and smoking cessation), and the risk reduction achievable after one or more risk-reducing therapies. It concludes by asking participants which risk reducing option they plan to pursue, reinforcing commitment to action.

The values clarification component helps individuals further clarify which risk reducing strategy they might pursue (i.e. which is most concordant with their values and would best allow them to adhere over time) by employing an explicit ranking and rating values clarification exercise. The ranking and rating exercise asks individuals to rank and rate attributes (e.g. benefit for other medical conditions, side effects, degree of difficulty in adherence for most people, cost, and effect on others) common to each of the treatment options in the decision aid [35]. It then summarizes their responses and asks them which risk reducing option they plan to pursue.

The coaching tool 1) outlines the benefits of participating with the provider in decision making about CHD risk reduction (e.g. learning about special issues unique to one’s health, getting information about resources that may help them accomplish their plan), 2) provides a menu of seven common barriers people have in talking with their provider about their plans (e.g. the provider decides the agenda, the provider uses too much medical talk, the provider does not acknowledge previous successes), and 3) provides audio clips matched with still photos of a narrator and patients talking about simple practical ways to overcome common barriers. Participants could explore any number of these barrier messages (none to seven) according to their interest and need. To increase relevance and engagement for individuals, the coaching tool includes photographs and voices representing diverse age and race/ethnicity groups.

At the end of the decision aid, all participants receive a summary of their Heart to Heart session that they can take to their provider to initiate discussion [32].

### Measures

#### Effect of the decision aid on knowledge and accuracy of risk perception

To assess participants’ knowledge of effective CHD prevention strategies, we conducted within-group comparisons of intervention participants’ knowledge at baseline and immediately post-decision aid. We asked, “What things do you think a person can do to lower his/her chances of heart disease?”, followed by a list that included strategies such as taking aspirin, blood pressure, and cholesterol medicine; stopping smoking; changing diet; and increasing physical activity. For each individual strategy, we measured the proportion of participants indicating “yes.” We then combined “yes” answers to aspirin, blood pressure and cholesterol medicine, and smoking cessation to create a composite reflecting knowledge of the “most effective strategy”.

We also conducted a within-group comparison of intervention participants’ accuracy of risk perception at baseline and post-decision aid. To measure accuracy of risk perception, we asked participants, “What do you think is your chance of developing heart disease in the next 10 years?” Answers included “less than 5%”; “6 to 10%”; “11 to 20%”; and “greater than 20%.” When participants’ calculated global CHD risk fell into the category they indicated on the survey, we categorized them as having an accurate risk perception.

#### Effect of the decision aid on decisional conflict and values clarity

To assess decisional conflict and values clarity, we conducted within-group comparisons of the intervention group at baseline and post-decision aid. To assess decisional conflict, we used a well-validated 16-item scale examining several features of conflicted decision making, including feeling uncertain, uninformed, unclear about one’s values, unsupported, or ineffective in making choices [36]. We summed and averaged responses for this scale, with scores ranging from 1 to 5. Scores less than 2 indicate low conflict and are associated with intent to make and implement a decision immediately.

To assess values clarity, we measured agreement with the statement, “My decision shows what is important to me.” Participants provided answers on a 5-point Likert scale dichotomized to strongly agree/agree versus all others.

#### Effect of the decision aid on patient-provider discussions and patients’ perceptions of their interaction with their provider

To determine the effect of the decision aid on patient-provider discussions, we assessed visit content in the intervention and control groups immediately using questions adapted from a prior study of prostate cancer screening [37].

We measured CHD discussions with the question, “Did you and your provider talk about lowering your chances of heart disease today?” If participants did report having a discussion, they reported who brought up the discussion (patient or provider), how much they participated in the discussion (a lot, some, a little, none), and who made the final clinical decision about CHD prevention (provider alone, provider and patient, patient). In this paper, we dichotomized participation in the discussion into any versus none and final decisions into shared (provider and patient) versus other.

To capture the effects of the coaching component of the decision aid, we measured patients’ perceptions of their interaction with their provider (e.g. “I feel my provider provided me with choices and options about lowering my chances of heart disease”; “My provider tried to understand how I see things before suggesting new ways to lower my chances of heart disease”) using the short-form 6-item Healthcare Climate Questionnaire. This scale is modified from the full 15-item measure [38] and has an alpha reliability of 0.82 (http://www.selfdeterminationtheory.org/questionnaires/10-questionnaires/81). Answers were measured on a 5-point Likert scale dichotomized to strongly agree/agree versus all others.

#### Participant intentions for CHD risk reduction across the decision aid

To assess the effectiveness of our decision aid overall and its components individually, we measured participants’ intentions to lower CHD risk among those eligible for intervention at various times across our study: at baseline and post-clinic visit in both the intervention and control groups, and additionally at multiple times during decision aid viewing in the intervention group (e.g. after the educational component, after the values clarification component, and after the completion of the entire decision aid). We assessed intention to lower CHD risk by asking, “Are you planning to lower your chances of heart disease?” We recorded participants’ intentions for risk reduction strategies singly and in combination by creating a composite “any effective CHD risk reducing strategy” variable that recorded intent to start any one of the effective risk reducing interventions that were a focus of our intervention (take aspirin, blood pressure, or cholesterol medication and/or to stop smoking).

#### Participants’ use of and feelings about the decision aid

To assess tool use, we electronically tracked total time spent with the decision aid; content of information received (including which informational links participants accessed); number of times participants recalculated their CHD risk; and which links participants followed about communicating with their provider. We did not solicit participants’ satisfaction with the time they spent with the tool, nor did we solicit free text comments about the decision aid. To summarize participants’ use of the decision aid, we calculated the mean and range of time spent with the tool and then stratified the mean amount of time spent with the tool by CHD risk level (0-5%, 6-10%, 11-20%, or >20%) and number of risk reducing options. We also calculated the mean number of times participants recalculated their CHD risk and again stratified by the number of CHD risk reducing options. Finally, we calculated frequencies for the number of participants accessing various informational links in the tool (including links for risk reducing options and for barriers to communicating with one’s provider) and for the number of participants ranking decisional attributes as “most important.”

To assess participants’ feelings about the decision aid, we calculated the frequencies of individuals who reported they agreed or strongly agreed with process statements such as “Heart to Heart helped me decide what’s important to me” and “Heart to Heart helped me make a decision that I could stick with”.

### Analyses

All analyses were conducted using SAS 9.2 Statistical Software (Cary, NC). To assess the success of randomization, we compared the baseline characteristics of subjects who received the intervention and those who did not. We adjusted all subsequent between-group analyses for identified appreciable differences.

To assess the effects of our decision aid on measures of decision making (including knowledge, accuracy of risk perception, decisional conflict, and values clarity), we conducted within-group comparisons. We used McNemar’s chi-square tests for dichotomous variables and paired t-tests for continuous variables and calculated the absolute difference from baseline to post-decision aid. All analyses accounted for the random effects of clustering of patients within providers.

To assess the effects of the decision aid on patient-provider discussions and patients’ perceptions of the visit, we used between group comparisons. We compared control and intervention groups’ responses using Rao-Scott chi-square tests. We additionally used mixed effects logistic regression models to calculate the absolute difference between groups, adjusting for both the baseline difference in education as a fixed effect and clustering within providers as random effect.

To assess the effect of the intervention on intent for adopting CHD risk reduction strategies, we again made between group comparisons post-clinic visit using Rao-Scott chi-square tests and, subsequently, mixed effects logistic regression models to adjust for the baseline differences in education and the natural effect of clustering of patients within providers.

To report on participants’ use of and feelings about the decision aid, we used descriptive analysis. We additionally used Spearman correlation coefficients to examine the relationship between time spent with the tool and education, comfort using a computer, CHD risk level, and the number of options for risk reduction.

### Sample size

Power was not calculated specifically for the secondary outcomes presented in this paper.

## Results

### Participant demographics

Our sample includes 24 eligible providers who had patients enrolled in the study and 160 eligible patients.

In general, patients were mostly white, male, had at least some college education and at least a good self-perceived health status. Mean age was 63. Most patients expressed a preference for shared decision making about CHD prevention (see Table 1). Groups were similar except that a higher proportion of individuals in the intervention group (98%) compared to the control group (82%) reported having at least some college education.

**Table 1:** Baseline participant characteristics

**Characteristic**	Total group N = 160*	**Control group N = 79**	**Intervention group N = 81**
Mean age	63	64	63
Female	28%	28%	27%
Race:			
White	86%	84%	88%
Black	10%	10%	10%
Education:			
At least some college	90%	82%	98%
CHD risk factors			
BP > 140/90	36%	37%	35%
TC/HDL ratio > 4	52%	51%	53%
Smoker	13%	13%	14%
Family history of CHD (age < 55)	23%	25%	21%
Mean CHD risk	11.3	11.4	11.2
Current risk reducing strategies:			
Blood pressure med	56%	61%	51%
Cholesterol med	29%	27%	31%
Smoking cessation	3%	4%	2%
Aspirin	44%	47%	42%
Diet low in saturated fat	58%	58%	58%
Exercise regularly	58%	54%	62%
Self-efficacy to lower at least 1 CHD risk factor	98%	96%	99%
Comfort using computer	91%	90%	93%
Any planned effective risk reducing strategy†	27%	25%	28%
Preferred participation in decision making about CHD:			
Share decision	86%	90%	82%
Do not share decision	14%	11%	19%
Accurately identified most effective strategies for risk reduction	53%	51%	54%
Accurately perceives CHD risk	24%	14%	34%
Decisional conflict	2.53	2.49	2.57
Decision consistent with values	69%	69%	68%

*160 participants at baseline; 3 missed Primary Study Visits and 3 missed follow-ups.

†This includes hypertension medicine, cholesterol medicine, smoking cessation, aspirin.

### Effect of decision aid on knowledge and accuracy of risk perception

After viewing the decision aid, participants in the intervention group had greater knowledge of effective CHD prevention strategies (see Table 2 for analyses adjusted for the effect of clustering of patients within providers). Knowledge of hypertension medication (+9 percentage points; adjusted p = .09), cholesterol medication (+17 percentage points; adjusted p < .01), and daily aspirin use (+27 percentage points; adjusted p < .0001) increased across the intervention, as did knowledge of all four effective CHD prevention strategies (hypertension and cholesterol medication, smoking cessation, and aspirin use; +28 percentage points, adjusted p < .0001). Moreover, the proportion of participants who accurately perceived their CHD risk also significantly increased (+33 percentage points; adjusted p < .0001).

**Table 2:** Effect of the Decision Aid on Knowledge, Accuracy of Risk Perception, and Values Clarity

	**Intervention group**		**Absolute difference**	Adjusted p-value*
	Baseline	Post-DA		
**Effect on knowledge**				
Identified “x” as strategy:				
Hypertension med	78%	87%	+9%	=.09
Cholesterol med	73%	90%	+17%	<.01
Smoking cessation	100%	96%	-4%	--
Aspirin daily	68%	95%	+27%	<.0001
Diet low in saturated fat	94%	96%	+2%	=.32
Exercise regularly	100%	99%	-1%	--
Accurately identified most effective strategies for risk reduction	54%	82%	+28%	<.0001
**Effect on accuracy of risk perception**				
Accurately perceives risk	34%	67%	+33%	<.0001
**Effect on decision**				
Decisional conflict†	2.57	1.94	-0.63	<.0001
Decision consistent with values‡	68%	83%	+15%	=.02

*Adjusted for clustering within providers.

†On alternate 0-100 scale: Baseline = 39.3; Post decision-aid = 23.5; absolute difference = -15.9; p < .0001.

‡From question: “My decision shows what is important to me”.

### Effect of decision aid on decisional conflict and values clarity

After viewing the decision aid, participants had significantly lower decisional conflict (baseline = 2.57; post-decision aid = 1.94; absolute difference -0,63; adjusted p < .0001) and crossed the threshold (e.g. decisional conflict between 2.5 and 2) believed to distinguish inaction from action. After viewing the decision aid, participants also tended to increase their self-reported values clarity (+15 percentage points, adjusted p = .02).

### Effect of decision aid on patient-provider discussions, and patients’ perceptions of their interaction with their provider

Participants in the intervention group were significantly more likely to have CHD prevention discussions with their provider than participants in the control group after adjustment for baseline differences in education and clustering of patients within providers (+31 percentaqe points; 95% CI 15 to 45 percentage points; adjusted p < .001). (See Table 3) Intervention participants were also more activated, with a tendency to more frequently raise the topic of CHD prevention (+28 percentage points; adjusted p = .02) and to participate more in the discussion (+28 percentage points; adjusted p = .01).

**Table 3:** Effect of decision aid on patient-provider discussions

	**Control group (n = 78)**	**Intervention group (n = 79)**	Absolute difference (95% CI)*	**Adjusted p-value†**
Had CHD discussion with their provider	58%	89%	31% (15% to 45%)	p < 0.001
Patient raised discussion	35%	63%	+28% (9% to 45%)	p = .02
Patient participation				
Any	51%	79%	+28% (9% to 45%)	p = .01
None	49%	21%	-28% (-45% to -9%)	
Who made final decision				
Shared decision	93%	84%	-9% (-27% to 10%)	p = .09
Not shared decision	7%	16%	+9% (-10% to 27%)	
**Patients’ perceptions of discussions and the health care visit**				
My provider provided me with choices and options about lowering my chances of heart disease.	76%	91%	+15% (-0.1% to 31%)	p = .02
My provider understands how I see things with respect to lowering my chances of heart disease.	86%	95%	+9% (-7% to 25%)	p = .21
My provider conveyed confidence in my ability to make changes regarding lowering my chances of heart disease.	77%	88%	+11% (-5% to 27%)	p = .15
My provider encouraged me to ask questions.	67%	78%	+11% (-4% to 27%)	p = .13
My provider listened to how I would like to do things.	71%	92%	+21% (6% to 37%)	p < .01
My provider tried to understand how I see things before suggesting new ways to lower my chances of heart disease.	69%	84%	+15% (-0.3% to 31%)	p = .05

*Adjusted for clustering within providers.

†Adjusted for baseline education and clustering within providers.

Intervention participants also tended to report better interactions with their provider, with improvements for the following 3 of 6 items from the Healthcare Climate Questionnaire: “My provider provided me with choices and options about lowering my chances of heart disease” (+15 percentage points; adjusted p = .02); “My provider listened to how I would like to do things” (+21 percentage points; adjusted p < .01); and “My provider tried to understand how I see things before suggesting new ways to lower my chances of heart disease” (+15 percentage points; adjusted p = .05). There were no significant differences in the proportion of patients reporting: “My provider understands how I see things with respect to lowering my chances of heart disease” (+9 percentage points; adjusted p = .21); “My provider conveyed confidence in my ability to make changes regarding lowering my chances of heart disease” (+11 percentage points; adjusted p = .15); and “My provider encouraged me to ask questions” (+11 percentage points; adjusted p = .13).

### Effect of decision aid on participant intentions for CHD risk reduction

Following their visit with their provider, participants in the intervention group were more likely to plan any of the effective CHD risk reducing strategies that were a focus of our intervention (e.g. aspirin, blood pressure medicine, cholesterol medicine, smoking cessation) than participants in the control group (+21 percentage points; 95% CI 5-37 percentage points) (see Table 4). Differences in intentions were greatest for intent to take aspirin (+19 percentage points; 95% CI -1 to 39 percentage points) and cholesterol medication (+30 percentage points; 95% CI 14 to 46 percentage points).

**Table 4:** Effect of decision aid and patient-provider discussions on patient intent for CHD risk reduction among those eligible for risk reduction

**Planned CHD interventions**	**Control group**		**Intervention group**					**Adj. absolute difference between control and intervention, *post visit *** (95% CI)*
	**Baseline (95% CI)**	**Post-visit (95% CI)**	**Baseline (95% CI)**	**Post-education (95% CI)**	**Post-values clarification (95% CI)**	**Post-coaching (95% CI)**	**Post-visit (95% CI)**	
Any effective CHD risk reducing Strategy† (n = 157)	25%	42%	28%	62%	61%	57%	63%	21%
	(15-36%)	(32-52%)	(21-36%)	(50-75%)	(49-73%)	(45-69%)	(49-77%)	(5% to 37%)
BP med, if HTN (n = 55 )	3%	29%	11%	65%	65%	37%	26%	-3%
	(0-11%)	(5-52%)	(0-24%)	(40-91%)	(45-86%)	(45-81%)	(7-45%)	(-30% to 25%)
Cholesterol med, if abnormal chol (n = 69)	12%	9%	5%	59%	59%	47%	39%	30%
	(0-26%)	(1-18%)	(0-13%)	(43-75%)	(44%-74%)	(28-67%)	(24-54%)	(14% to 46%)
Smoking cessation, if smoking (n = 21)	60%	50%	82%	70%	80%	80%	80%	30%
	(25-95%)	(8-92%)	(56-100%)	(35-100%)	(50-100%)	(50-100%)	(51-100%)	(-16% to 76%)
Aspirin, if CHD risk >6% and no contra-indication (n = 140)	12%	24%	14%	40%	40%	47%	43%	19%
	(8-17%)	(14-33%)	(6-21%)	(27-54%)	(29-52%)	(31-63%)	(24-62%)	(-1% to39%)
Diet low in saturated fat, all (n = 157)	23%	40%	20%			46%	29%	-11%
	(12-34%)	(29-51%)	(12-28%)	--	--	(44-65%)	(16-42%)	(-27% to 6%)
Exercise regularly, all (n = 157)	35%	54%	34%	--	--	56%	53%	-1
	(24-47%)	(39-69%)	(23-45 %)			(42-69%)	(44-62%)	(-17 to 16)

*Adjusted for random effects of clustering within provider.

†Includes strategies that were the focus of our intervention: aspirin, blood pressure medicine, cholesterol medicine, smoking cessation.

Changes in intentions to start any effective CHD risk reducing strategy were most pronounced after the education component of the decision aid (+34 percentage points), and changed little with additional viewing on the values clarification and coaching components of the decision aid. These patterns were fairly consistent for intent to start individual CHD prevention therapies. However, there was a trend for reduced intent to take hypertension medication following the provider visit.

### Participants’ use of the decision aid

Participants spent an average of 12 minutes with the Heart to Heart decision aid (standard deviation 7 minutes; range: less than one minute to 45 minutes). 6% spent 0-5 minutes, 30% spent 6-10 minutes, 34% spent 11-15 minutes, 18% spent 16-20 minutes, and 11% spent 21 or more minutes. Time spent with the tool did not differ by CHD risk level (10-year predicted CHD risk <0-5%, 6-10%, 11-20%, or >20%). It did, however, vary by education (Spearman rho -0.29) and comfort using the computer (Spearman rho 0.34). Participants recalculated their CHD risk an average of two times (range 1-6), and appropriately recalculated their risk more times as their number of options for CHD risk reduction increased (participants with 2 options recalculated an average of 2.6 times and those with 4 options recalculated an average of 3.5 times). Over 20% of participants accessed links providing additional information about the effects of aspirin, cholesterol medicine, diet, and exercise. Only 13% and 3% accessed informational links about blood pressure medicine and smoking, respectively.

Participants indicated different values that were important to them with respect to CHD prevention, with “level of difficulty” as the most important (51%), followed by “other health benefits” (31%) and “side effects” (12%). Participants most commonly accessed the following links for barriers to communicating with their provider: “I respect my provider, so I agree to his/her plans even though I know I won’t stick to them” (27%); “My provider recommends medicine, but I want to change my lifestyle” (21%); and “I want different treatment options than my provider recommends” (14%). Other links were accessed less frequently.

### Participants’ feelings about the decision aid

Most participants found the decision aid easy to use (92%); easy to understand (99%); and said it personalized information for them (90%). Almost 80% of participants reported the decision aid helped them decide what was important to them and helped them make a decision to which they could adhere. Seventy-one percent reported the printout they received after viewing the decision aid would help them to start a conversation with their provider.

## Discussion

We developed a CHD prevention intervention that included a decision aid that participants found easy to use and understand and said helped them to make decisions about medications to which they could adhere. The decision aid increased participants’ readiness to make CHD prevention decisions by increasing knowledge, accuracy of risk perception, and values clarity, and by decreasing decisional conflict. The decision aid further increased the proportion of CHD prevention discussions with providers, patients’ perceptions that at least some features of patient-provider interactions were more favorable, and patients’ intentions for any effective risk reducing strategy. The majority of the impact of the decision aid on patients’ intent to start CHD prevention interventions appeared to result from the educational component of our decision aid.

Our findings are consistent with prior systematic reviews, which suggest that decision aids improve patient knowledge, increase the likelihood of making decisions, and improve patient-provider discussion [27,39]. Our research, however, suggests that much of the impact of decision aids on intent to start any medical therapy for CHD prevention may result largely from education about CHD risk (with implicit values clarification) alone. We were unable to detect any changes in intent for specific therapies with explicit clarification of values through our ranking and rating values clarification process. Further, coaching to improve the patient-provider interaction and provider visits did not appear to affect intent (except for a trend toward reduced hypertension medication following the provider visit). However, this needs replication in large comparative effectiveness trials or studies of other design that randomize the order of decision aid components.

Moving forward, researchers should consider how to make interventions more effective in promoting use and adherence to effective treatment strategies. For instance, researchers might include information about drug formulary coverage into decision aids so that patients can make even more informed choices about the best possible risk reducing strategies: those consistent with personal preferences and values and with few barriers to adherence. They might also incorporate messages that address possible misconceptions for those who make no plans to pursue any of the available and effective treatment options. In our study, we delivered such messages as tailored messages after the clinical visit (see Additional file 1). However, incorporation of such messages into the decision aid might improve clinical efficiency by allowing completion of decision making at the point of care. Finally, researchers might consider strategies for maximizing information delivery. Users in our study infrequently (~20%) accessed links for additional information (e.g. exact bleeding rates with aspirin) which may have influenced decision making. Similarly, relatively few participants accessed each coaching message (<30% for any given message), raising the question of whether decision aids should be designed to force individuals to proceed through all parts of a decision aid, or whether patients should be able to choose the information they want to view.

Future research should also give attention to the best ways to implement decision aids such as ours into clinical and public health practice. CHD prevention decision aids are not only responsive to recent national campaigns (e.g. The Million Hearts Campaign) that call for the use of internet technology to help identify and support patients at elevated risk of CHD [40], but they also have the potential to support the mission of burgeoning health care delivery mechanisms (e.g. accountable care organizations) that call for increased quality and efficiency and reduced healthcare costs [41]. To ensure decision aids’ maximal impact, however, the success of various implementation strategies must be studied. At present, there are multiple approaches to decision aid implementation (e.g. via the web at home or in community settings, in the office prior to the clinical visit, and integrated into the clinical visit in full or abbreviated form), but evidence is lacking to determine which approach results in the best outcomes [42,43]. Further, very little work has been done to explore how integrating decision aids into patient portals with prompts from the electronic health records would impact clinical outcomes. Investigating these topics could yield significant insights and benefits for care delivery.

In considering our findings, readers should consider the following limitations. First, our sample size was determined by our ability to detect differences in our main study outcome (CHD risk reduction), not the secondary outcomes reported in this paper [32]. Future studies should be powered specifically to look at decision making outcomes. Second, baseline differences in education among intervention and control patients raise the possibility of unmeasured confounding. Third, appropriate measures of coaching success are unknown. We chose to measure coaching success using the presence of CHD discussions and a validated scale of patients’ perceptions of their interaction with their provider. Other measures of coaching may yield additional or different insights. Fourth, our examination of the impact of various components of the decision aid should be viewed as preliminary only. Subgroups were small and our study design did not allow assessment of the independent effects of decision aid components. Fifth, the patient sample was mostly white, highly educated, and had high self-efficacy. Results, therefore, may not generalize to more diverse patient populations.

## Conclusions

Limitations aside, this study shows that decision aids can play an important role in improving decisions about CHD prevention and increasing CHD discussions and intent to reduce CHD risk. Additional insights might be gained by studying the effects of decision aids on more diverse populations, with additional measures of coaching success, and more diverse strategies for decision aid implementation.

## Competing interests

The authors declare that they have no competing interests.

## Authors’ contributions

SS conceived of the study and its design, participated in data analysis, and wrote the manuscript. LD participated in acquisition of data, data analysis, and manuscript drafting. MP, BR, TK, and RS participated in study design and reviewed the manuscript critically for intellectual content. SB participated in study design and data analysis. JC participated in data analysis. ZG participated in study design and performed data analysis. All authors read and approved the final manuscript.

## Pre-publication history

The pre-publication history for this paper can be accessed here:

http://www.biomedcentral.com/1472-6947/14/14/prepub

## Supplementary Material

Additional file 1 Messages for Individuals Choosing No Options for CHD prevention. Click here for file
