# Supplementary material for: The effect of a decision aid intervention on decision making about coronary heart disease risk reduction: secondary analyses of a randomized trial
Source: BMC Med Inform Decis Mak. 2014 Feb 28;14:14. doi: 10.1186/1472-6947-14-14 (PMC3943405; doi:10.1186/1472-6947-14-14)
Supplement: Additional file 1 — Messages for Individuals Choosing No Options for CHD prevention. [file 1472-6947-14-14-S1.docx]

**Additional File 1. Messages for Individuals Choosing No Options for CHD prevention**

The messages below were delivered as tailored messages to participants choosing no option for CHD prevention. They might be more effective if delivered as part of the decision aid as their goal is to address possible misconceptions.

| **Reason for Choosing No Medicine** | **Message** |
| --- | --- |
| Chances of heart disease aren’t high enough | Many people whose chances of heart disease are high don’t feel that their chances are high enough to make a change.  What they forget is that heart disease is the number one killer in the United States.  •It kills more people each year than lung cancer, colon cancer, breast cancer, and prostate cancer combined.  •This year, 700,000 Americans will have their first heart attack. 224,000 will die from it.  •82% of people who have a heart attack do not have warning signs (such as repeated episodes of chest pain).  Therefore, it is a good idea for people with high chances to find a risk-reducing strategy that works for them! |
| Need more time for decision making | Taking the time to choose a plan that is right for you is important so that you can stick with whatever plan you choose. But sometimes people delay making a plan because they don’t have enough information about their options, aren’t sure what’s important to them, or need some input from their doctor.  You’ve now had some time to think over a plan, so ask yourself:  • Am I ready to commit to a plan?  • Do I have enough information?  • Am I sure what’s important to me?  • Do I need some input from my doctor?  If you find you need more information:  • Consider contacting the American Heart Association:  * [www.americanheart.org](http://www.americanheart.org)  * 1-800-242-8721  If you still aren’t sure what’s important to you:  • Visit the Ottowa Personal Decision Guide at http://decisionaid.ohri.ca/decguide.html  If you need some input from your doctor:  • Call your doctor.  Schedule an appointment with your doctor. The number is xxx. |
| Don’t like the idea of medicine | Many people don’t like the idea of taking medicine unless they “have to”. That’s generally a good idea. But there may come a time in the future when other factors outweigh your feelings about taking medicine.  Ask yourself: “When would I be willing to take a medicine?”    For example, would I be willing to take it if:  • I started developing chest pain?  • My chances of heart disease increased (note: this happens as you age)?  • I changed my lifestyle and my chances of heart disease didn’t change?  • My doctor told me it’s the best thing for me?  Asking yourself these questions now can help you make a plan that’s right for you. |
| Can’t find an option that meets my needs | One of the great things about lowering your chances of heart disease is that there are many options to do it! With a little effort and help from your doctor, you can find a medicine or smoking cessation plan that meets your needs, finances, and lifestyle.  If you are worried about the amount of difficulty involved in making a change, consider medicine over lifestyle changes such as quitting smoking.  If you are worried about cost, consider aspirin or free quitlines (if you are a smoker).  If you are worried about side effects, consider one of the many blood pressure medicine options or counseling to quit smoking.  There’s a good option out there for you! |
| Intervening will reduce my enjoyment in life | Many people think that taking a medicine or stopping smoking would limit their enjoyment of life. These people often don’t consider how much their enjoyment would be reduced if they had a heart attack.  Heart attacks can result in:  • Hospitalizations  • Medical bills  • Long-term symptoms such as fatigue, shortness of breath, chest pain, and leg swelling  These things can get in the way of staying active and being able to do the things you want to do.  Ask yourself:  • Have I thought about what might happen if I don’t lower my chances of heart disease?  • Is my enjoyment now worth the chances of a heart disease in the future?  If you answered no to either question, you might want to reconsider a plan to lower your chances of heart disease. Give your doctor a call to discuss your options. |
| Medicine isn’t a priority | Many things get in the way of lowering one’s chances of heart disease. Sometimes it’s okay for these things to take priority. However, sometimes they take priority simply because we haven’t stopped to consider all of the facts.  To help you decide whether you want to make heart disease a priority right now, consider:  • Is heart disease more or less important to you than the other things?  • Are you willing to risk the chance of a heart attack or sudden death?  • Do you have the resources you need to lower your chances of heart disease?  If heart disease is a priority right now, contact your doctor.  If you have other things you need to take care of right now, set a time to reassess your priorities in the next 2-3 months. |
